# Bridging the Gap in United Nations Environment Programme’s Report: The Potential of Nudges to Reduce Plastic Pollution and Health Risks

**DOI:** 10.7759/cureus.60169

**Published:** 2024-05-12

**Authors:** Mira Namba, Yudai Kaneda, Tetsuya Tanimoto, Masaki Takebayashi

**Affiliations:** 1 Medicine, Keio University, Tokyo, JPN; 2 Medicine, Hokkaido University, Sapporo, JPN; 3 Internal Medicine, Navitas Clinic, Tokyo, JPN; 4 Sociology, Aomori University, Aomori, JPN

**Keywords:** recycling, pet bottles, nudge, japan, health issue

## Abstract

Plastic pollution is increasingly becoming a threatening problem worldwide, with highlighted health risks associated with plastic waste incineration. Among the immediate measures to address this problem, proper recycling of polyethylene terephthalate (PET) bottles is a pertinent strategy. Considering the urgent need for administrative-led reforms, we assessed the separation rates of PET bottle caps and labels by 125 administrative staff in a government office of a prefectural in Japan during a four-day period; only 59.3% (48/81) of the pet bottles had both caps and labels removed and properly separated to each trash can.

One potential solution for the low separation rate is the use of nudges, which are effective methods for promoting behaviors such as healthy actions. Since both health and environmentally conscious behaviors involve choices across different time points, leveraging insights from nudges developed in the field of health behavior to environmental behaviors is considered crucial, even from a health promotion perspective.

## Editorial

In May 2023, the United Nations Environment Programme (UNEP) released an urgent report on the significant burdens posed by plastic pollution and a roadmap for the world to address [1]. The report highlighted health risks associated with plastic waste incineration, including lung disorders, abnormal sperm formation, and increased obesity due to endocrine-disrupting substances. Of particular concern is the potential serious impact on the health of women exposed to bisphenol A (BPA), one of the endocrine-disrupting substances, which may lead to conditions such as polycystic ovary syndrome, obesity, recurrent miscarriage, endometrial hyperplasia, and infertility [2]. A recent report by Marfella et al. found patients in whom microplastics and nanoplastics were detected within the atheroma were at 4.53 times higher risk for myocardial infarction, stroke, or death from any cause than those in whom these substances were not detected [3]. Although the UNEP report reflects differences in opinions among countries on various crucial aspects, such as regulatory measures and financial support, there is a lack of concrete solutions.

In Japan, the Plastic Resource Circulation Promotion Law was enacted in 2022 to encourage initiatives from the design of plastic products to their disposal, collection, and recycling, aiming to promote the circularity of plastic resources. Among the immediate measures consumers can implement, the proper recycling of PET bottles stands out as a pertinent strategy. However, a consumer awareness survey on PET bottles, conducted nationwide simultaneously with the enforcement of this law, revealed a recycling rate of approximately 36.2% indoors, including workplaces, compared to 74.6% at home [4].

Moreover, amidst administrative-led reforms, there was insufficient information about the waste separation habits of administrative staff. Therefore, during a four-day period, the separation rates of PET bottle caps and labels in waste bins in the tea rooms of a prefectural government office were assessed. Ethical considerations were confirmed by the authors using the "Ethical Checklist for Utilizing Behavioral Insights Such As Nudges" developed by the Behavioral Sciences Team (the Japanese government’s Nudge Unit) [5]. Approval from a prefectural government and communication to staff via the intranet were obtained before implementation.

The results are presented in Table 1. Out of the total 81 discarded PET bottles by 125 administrative staff during the period, 48 (59.3%) had both caps and labels removed and properly separated. Despite the presence of administrative staff with a strong sense of normative consciousness, the compliance rate was lower than that of individuals separating waste at home.

**Table 1:** Status of PET Bottle Separation PET: polyethylene terephthalate

	Correctly separated (n=48)	Not correctly separated (n=33)	Total (n=81)
Day 1	17 (21.0%)	11 (13.6%)	28 (34.6%)
Day 2	13 (16.0%)	9 (11.1%)	22 (27.2%)
Day 3	9 (11.1%)	9 (11.1%)	18 (22.2%)
Day 4	9 (11.1%)	4 (4.9%)	13 (16.0%)

This raises concerns about the situation when waste separation is left to individual autonomy, emphasizing the need for appropriate interventions. One potential solution is the use of nudges, a concept originating in the field of behavioral science. Nudges are effective methods for promoting behaviors, such as healthy actions. Since the COVID-19 outbreaks, nudges have been applied to improve COVID-19 vaccination rates through text message reminders and to encourage the use of hand sanitizers through visibility nudges [6]. Both health and environmentally conscious behaviors involve choices across different time points. Implementing social or timely nudges in the field of waste separation, such as Norway's successful strategy of sending letters to compare household recycling practices, which increased recycling rates by 2% within seven months [7], demonstrates the effectiveness of these interventions.

Of note, while nudges are effective for immediate behavioral change, it is crucial to recognize that they do not address the fundamental reasons why individuals may fail to sort waste properly, and there are limitations to their long-term sustainability [8]. Additionally, implementing nudges must also consider ethical and social issues related to individual rights [9]. Bearing these points in mind, it is essential to combine education with nudges to simultaneously reform individual awareness and carefully pursue behavior stabilization through cautious interventions [10].

Despite extensive research on public awareness of waste separation in Japan, studies on its practical implementation are notably scarce, contrasting with emerging reports of nudge strategies from diverse countries. Leveraging insights from nudges developed in the field of health behavior, applying them to environmentally conscious actions, and demonstrating effective interventions are also crucial from a health promotion perspective. Thus, further advancing research in this field and accumulating these insights not only benefits Japan but also has the potential to be applied in similarly nudge-cautious countries such as Hungary [11], contributing to the protection of the environment and human health worldwide.
